# Proteomics of the organohalide-respiring Epsilonproteobacterium *Sulfurospirillum multivoran*s adapted to tetrachloroethene and other energy substrates

**DOI:** 10.1038/srep13794

**Published:** 2015-09-21

**Authors:** Tobias Goris, Christian L. Schiffmann, Jennifer Gadkari, Torsten Schubert, Jana Seifert, Nico Jehmlich, Martin von Bergen, Gabriele Diekert

**Affiliations:** 1 Department of Applied and Ecological Microbiology, Institute of Microbiology, Friedrich Schiller University, 07743 Jena, Germany; 2 Department of Proteomics, Helmholtz Centre for Environmental Research – UFZ, Permoserstr. 15, 04318 Leipzig, Germany; 3 Institute of Animal Science, Hohenheim University, 70593 Stuttgart, Germany; 4 Aalborg University, Department of Biotechnology, Chemistry and Environmental Engineering, Sohngårdsholmsvej 49, 9000 Aalborg, Denmark; 5 Department of Metabolomics, Helmholtz Centre for Environmental Research – UFZ, Permoserstr. 15, 04318 Leipzig, Germany

## Abstract

Organohalide respiration is an environmentally important but poorly characterized type of anaerobic respiration. We compared the global proteome of the versatile organohalide-respiring Epsilonproteobacterium *Sulfurospirillum multivorans* grown with different electron acceptors (fumarate, nitrate, or tetrachloroethene [PCE]). The most significant differences in protein abundance were found for gene products of the organohalide respiration region. This genomic region encodes the corrinoid and FeS cluster containing PCE reductive dehalogenase PceA and other proteins putatively involved in PCE metabolism such as those involved in corrinoid biosynthesis. The latter gene products as well as PceA and a putative quinol dehydrogenase were almost exclusively detected in cells grown with PCE. This finding suggests an electron flow from the electron donor such as formate or pyruvate via the quinone pool and a quinol dehydrogenase to PceA and the terminal electron acceptor PCE. Two putative accessory proteins, an IscU-like protein and a peroxidase-like protein, were detected with PCE only and might be involved in PceA maturation. The proteome of cells grown with pyruvate instead of formate as electron donor indicates a route of electrons from reduced ferredoxin via an Epsilonproteobacterial complex I and the quinone pool to PCE.

Halogenated hydrocarbons of anthropogenic origin have been widely used, e.g. as solvents or pesticides, during the last two centuries and are among the major pollutants in the environment. Additionally, organohalides are formed in biogeochemical processes and are therefore abundant in nature since billions of years. As a consequence, it is not surprising that microorganisms have adapted to exploit these compounds as nutrients in the course of the evolution^1^. Under anoxic conditions, halogenated hydrocarbons may be dehalogenated by reductive dehalogenation that is performed by bacteria and can be coupled to ATP synthesis via electron transport phosphorylation (organohalide respiration). Organohalide-respiring bacteria (OHRB) are found in phylogenetically diverse bacterial phyla like the *Proteobacteria*, *Firmicutes* and *Chloroflexi*^2^,^3^ and are classified as obligate or non-obligate organohalide respirers.

The Epsilonproteobacterium *Sulfurospirillum multivorans* (formerly *Dehalospirillum multivorans*) was isolated from activated sludge of a waste water treatment plant^4^. *S. multivorans* is a non-obligate OHRB, which reductively dehalogenates tetrachloroethene (PCE) and trichloroethene (TCE) to *cis*-1,2-dichloroethene (*c*DCE)^5^. The organism is able to utilize several different electron donors (e.g. hydrogen, formate, pyruvate, lactate) and a broad range of electron acceptors (e.g. fumarate, nitrate and many others besides PCE) for anaerobic respiration^4^,^6^. The key enzyme of PCE respiration is the PCE reductive dehalogenase (PceA), a corrinoid and FeS cluster containing enzyme, which is attached to the periplasmic face of the cytoplasmic membrane most probably via the putative membrane-anchor protein PceB^7^,^8^. The genome of *S. multivorans* is described and contains 3,233 protein-coding sequences^6^. Among them, a 50 kbp gene region was identified, which is not present in non-organohalide respiring *Sulfurospirillum* species. This region contains, among others, the *pceAB* operon, a second reductive dehalogenase gene cluster, genes for two-component regulatory systems, a set of genes encoding proteins involved in corrinoid biosynthesis and several genes encoding for proteins with putative accessory functions^6^. The corrinoid cofactor of PceA, a norpseudo-B_12_^9^, is synthesized *de novo* in *S. multivorans*^10^. This special type of corrinoid cofactor is not known to be produced by any other bacterium. Unlike in other OHRB, two genes encoding a putative quinol dehydrogenase were identified adjacent to the reductive dehalogenase structural genes^6^. The corresponding gene products show similarities to the NapGH quinol dehydrogenase of *Wolinella succinogenes*, which transfers electrons from the menaquinone pool to the terminal reductase in nitrate respiration, the periplasmic NapA^11^. In the absence of PCE as terminal electron acceptor during several transfers of *S. multivorans*, the *pceA* expression gradually ceased^12^. Based on this result, the generation of *S. multivorans* cells lacking PceA is possible. Recently, the quinol dehydrogenase genes in close proximity to the *pce* gene cluster were shown to undergo a similar transcriptional down-regulation in the absence of PCE^6^.

The PCE-dependent transcriptional up-regulation of the mentioned genes raised the need to validate these findings on the protein level and to compile global proteomic profiles specific for different electron donors and acceptors. The versatile metabolic capacities of *S. multivorans* allowed for a comprehensive comparison of proteome profiles originating from cultivations with pyruvate or formate as electron donors and either PCE, fumarate or nitrate as electron acceptors. This analysis allows for conclusions on the protein inventory involved in the PCE respiratory chain and in maturation of the required cofactors and proteins.

## Results

### Protein identification and relative quantification

**Figure 1:**
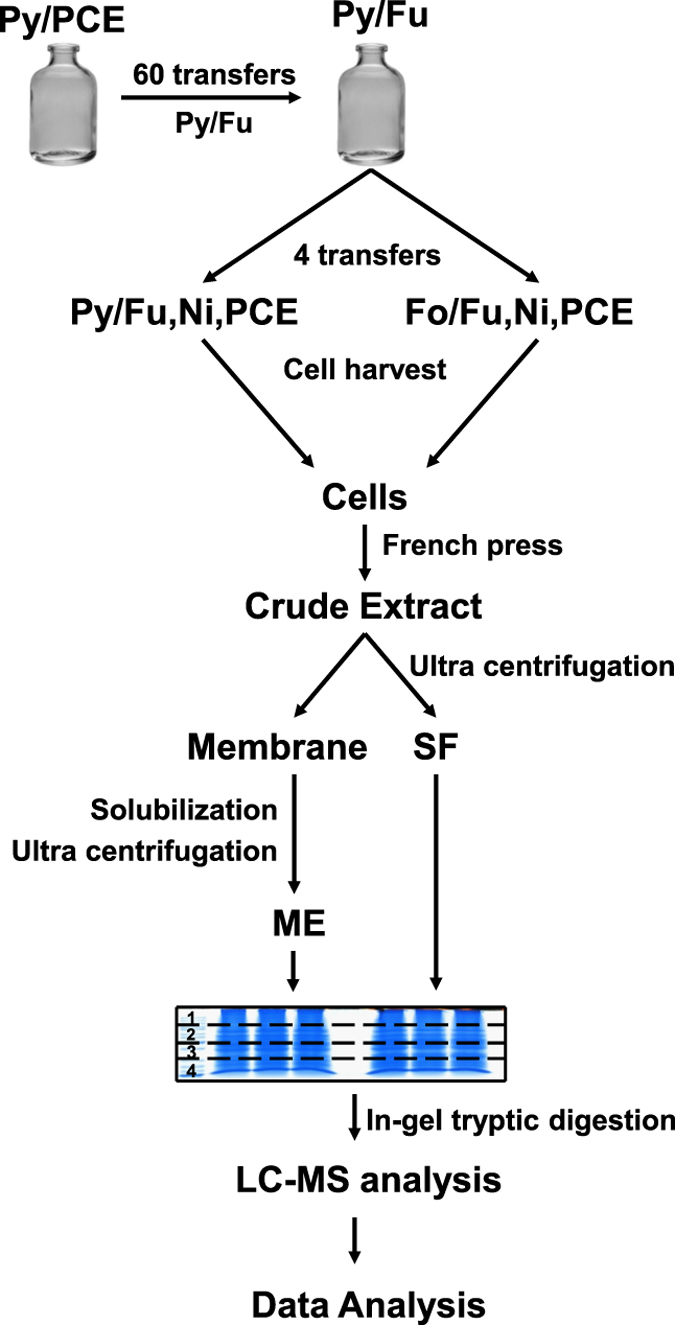
Experimental design of the differential proteomics approach used in this study. Grown cultures of *S. multivorans* were harvested and prepared for LC-MS measurement after repeated transfers (10% inoculum) on the desired substrates (Py, pyruvate; Fo, formate; Fu, fumarate; Ni, nitrate; PCE, tetrachloroethene) to get rid of any residual amounts of previously applied electron acceptors. The initial 60 transfers on Py/Fu were carried out to achieve down-regulation of PCE respiration in *S. multivorans* cells^12^. Membrane protein extract (ME) and soluble protein fraction (SF) were subjected to 1D-SDS PAGE. After in-gel digestion, peptide lysates were analyzed by LC-MS.

Diverse substrate combinations were applied to analyze the proteome profile of *S. multivorans*. Pyruvate and fumarate (Py/Fu) was used as the standard condition with the following electron donor/acceptor combinations for comparison: pyruvate with nitrate (Py/Ni) or PCE (Py/PCE); formate with fumarate (Fo/Fu), PCE (Fo/PCE) or nitrate (Fo/Ni). After harvest and disruption of cells, samples were fractionated into soluble fraction (SF) and membrane extract (ME). Subsequently, the proteomes of SF and ME were analyzed by LC-MS (Fig. 1). Between 689 and 918 proteins were identified in a single LC-run. In total, 1,716 distinct proteins were identified under at least one cultivation condition (Table S1), which results in 53% coverage of the annotated 3,191 non-redundant protein-coding sequences of *S. multivorans.* Among all conditions, we were able to quantify 616 proteins. Of these, 241 proteins were predicted to contain between one and 15 putative transmembrane helices calculated by an *in silico* topology analysis with Phobius^13^. The counts for those putative membrane proteins were from twofold (Py/Ni) to fivefold (Fo/Ni) higher in the membrane extract compared to the soluble fractions.

**Figure 2:**
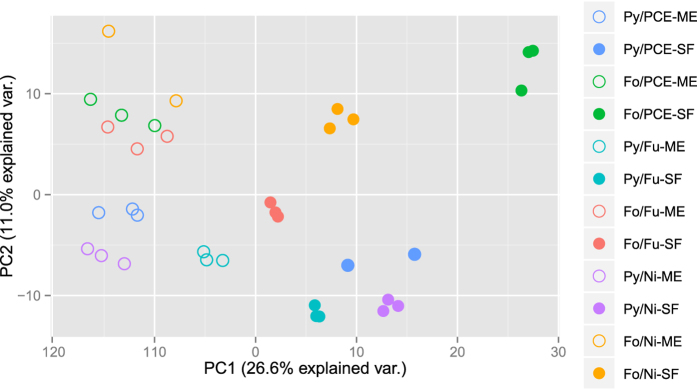
Principal component analysis of proteome profiles. Cultivation conditions are indicated by different colors, membrane samples are marked with open circles, soluble fractions by filled circles. The first two letters represent the electron donor: Fo, formate; Py, pyruvate, followed by the electron acceptor Fu, fumarate; Ni, Nitrate and fraction ME, membrane extract; SF, soluble fraction. Proteins quantfied in at least half of all measurements were included in the analysis (n = 672). The conditions Fo/Ni-ME and Py/PCE-SF include two points only, as one of the biological triplicates of each were identified as outliers using statistical analysis described in the methods section and were therefore not considered in the data analysis.

A principal component analysis (PCA) of quantified proteins was performed as indicator of the variance in the dataset. We observed that the biological replicates cluster appropriately, membrane extract and the soluble fractions can be distinguished and the dataset revealed reasonable reproducibility (Fig. 2).

### Overview on the PCE-induced protein pattern

Proteome analysis of *S. multivorans* grown with PCE, fumarate or nitrate as electron acceptors led to the identification of 119 proteins exclusively present in PCE-grown cells (Table S2). From these 119 proteins, 25 were quantified reliably (i.e. quantified in at least 50% of the biological replicates). Another 16 proteins were quantified in all samples with at least 2-fold higher protein abundance in Py/PCE and Fo/PCE cells compared to nitrate or fumarate (Fig. S1 and Table S3). The protein abundance of 28 proteins was significantly lower (at least 3-fold, p < 0.05 or not identified, Table S3) in Py/PCE and Fo/PCE cells when compared to the corresponding fumarate- or nitrate-grown cells.

### Proteins of the OHR region

**Figure 3:**
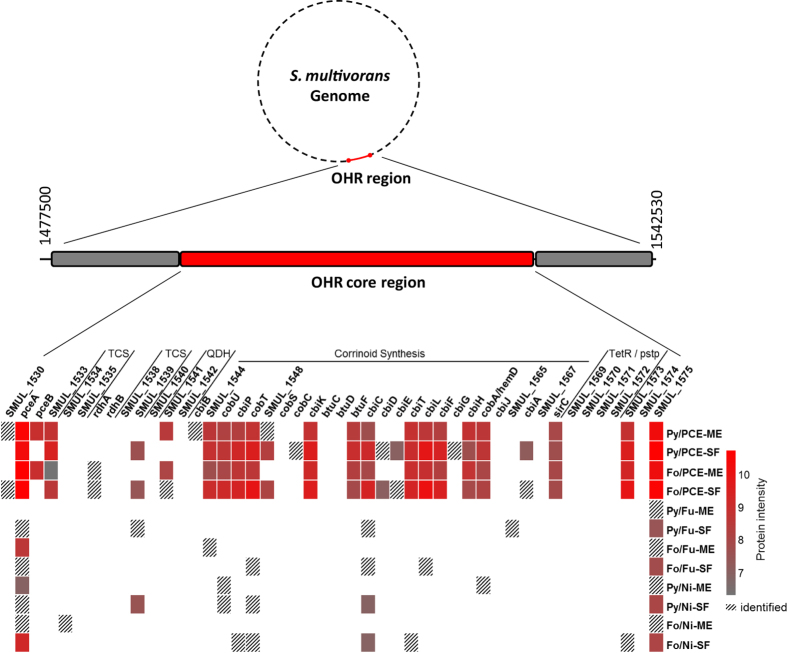
Identified gene products of the OHR core region as detected by proteomic analyses. Gene names or locus tags are given at the top of the protein intensity pattern. Each square correlates to a given gene product identified or quantified under a given cultivation condition (at the right). For quantified proteins the protein intensity is provided (color code at the far right, normalized and logarithmized average of top 3 peptide area as described in the methods section), proteins identified but not quantified are marked with shaded squares. Genes which functionally belong together are grouped by flanking lines and given the following abbreviations: TCS, two component regulator system; QDH, quinol dehydrogenase; TetR/pstp, *tetR* pseudogene disrupted by transposase.

Previously, an approximately 50 kbp large region was identified in the genome of *S. multivorans* with no similar genes found in closely related *Sulfurospirillum* spp. genomes, encoding proteins directly (for example PceA) or indirectly (e.g. for the corrinoid cofactor biosynthesis) involved in organohalide respiration. This region, ranging from SMUL_1516 to SMUL_1596, was therefore termed OHR (organohalide respiration) region^6^. Of the proteins encoded in the OHR region, 46 have a functional correlation to organohalide respiration and are located in the OHR “core” region (SMUL_1530 to SMUL_1575). Of them, 29 were identified and 24 were quantified with PCE as electron acceptor in at least one of the four samples (Py/PCE-ME, Py/PCE-SF, Fo/PCE-ME, Fo/PCE-SF; Fig. 3). Products of the genes which are located upstream or downstream of this OHR “core” region were not detected. In cells grown without PCE as electron acceptor, 14 of the 46 proteins of the “core” OHR region were detected in at least one of the eight samples. Only four of these 14 proteins could be quantified. Of these, the genes encoding PceA (SMUL_1531), CbiC (SMUL_1555) and a putative FMN-binding protein (SMUL_1575), were detected in significantly higher levels in PCE-grown cells (at least 50-times higher with p < 0.05 or not present without PCE; CbiC with p = 0.06 in Py/PCE-SF vs PyNi-SF, Fig. 3, Table S3) while a two-component response regulator (SMUL_1539) was not differently produced. PceA is subjected to a long term regulation^12^ and the other proteins encoded in the OHR core region follow presumably a similar pattern, with no or very low amounts detected in long-term cultivated cells without PCE (Fig. 3).

**Figure 4:**
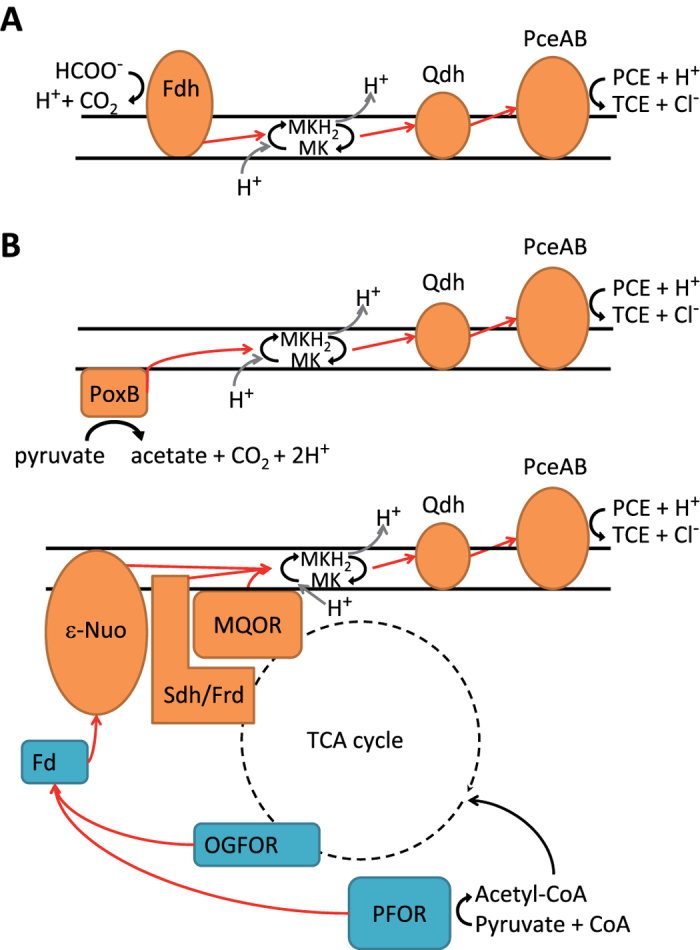
Overview of PCE-dependent catabolism in *S. multivorans.* (**A**) Formate-dependent PCE respiratory chain. (**B**) Pyruvate-dependent PCE respiratory chain, top: directly across the membrane employing quinone-reducing pyruvate dehydrogenase, bottom: cytoplasmic route of pyruvate oxidation via TCA cycle intermediates and ferredoxin. Abbreviations: Fdh, formate dehydrogenase; PoxB, pyruvate (quinone) dehydrogenase; Qdh, putative quinol dehydrogenase; PceAB, PCE reductive dehalogenase; PFOR, pyruvate:ferredoxin oxidoreductase; MQOR, malate:quinone oxidoreductase; Sdh/Frd, succinate dehydrogenase/fumarate reductase; OGFOR, 2-oxoglutarate:ferredoxin oxidoreductase; Fd, ferredoxin; ε-nuo, epsilonproteobacterial complex I; red arrows depict flow of electrons.

The PCE reductive dehalogenase PceA, the key enzyme of the PCE respiratory chain (Fig. 4), represents the most abundant protein encoded on the OHR region (Fig. 3) and is in general among the most abundant proteins in *S. multivorans* cells grown with PCE as electron acceptor (Table S4). In PCE-grown cells, PceA was present in an at least 32-fold higher amount (see Table S3). These data are in accordance to immunoblot analysis of the PceA protein and PceA activity of cell extracts measured in the same samples (Fig. S2, Table S4). PceB, the putative membrane anchor of PceA^8^, predicted to contain two transmembrane helices, was found exclusively in Py/PCE-ME and Fo/PCE-ME (Fig. 3). The second reductive dehalogenase (Fig. 3) was identified at low levels in Fo/PCE-cells only, while RdhB, the corresponding putative membrane anchor was not detected. Downstream of the second *rdhA* cluster, two genes are located which encode a putative quinol dehydrogenase (Fig. 3). One of the corresponding products, the putative periplasmic FeS cluster containing subunit (SMUL_1541) could be quantified exclusively in the membrane extracts of Fo/PCE and Py/PCE-grown cells. The membrane-integral subunit of the quinol dehydrogenase (SMUL_1542) was not detected, which might be attributed to its tight interaction with the membrane.

The following proteins encoded in the OHR region were exclusively identified in cells cultivated with PCE as electron acceptor. Directly upstream of *pceA*, an alkylhydroperoxidase-like-protein is encoded by SMUL_1530. This protein was identified in Py/PCE-ME and Fo/PCE-SF only. A small protein with unknown function (SMUL_1533) is encoded downstream of the *pceAB* gene cluster and was quantified in all PCE-samples. It shows low similarities to proteins involved in FeS cluster maturation (21% amino acid sequence identity to the characterized *E. coli* IscU^14^). SMUL_1533 was quantified in all PCE-samples. It showed protein values (normalized and logarithmized average of top 3 peptide area) of 9.4 in Py/PCE-SF and 8.5 in Fo/PCE-SF, the 3 to 10-fold compared to the membrane extracts (Fig. 3). The amount of IscSU/NifSU (SMUL_2994-2995), the epsilonproteobacterial FeS cluster biosynthetis proteins^15^ is not significantly altered in the presence of PCE. A small putative membrane protein (SMUL_1540) with three predicted transmembrane helices was not identified. Downstream of each reductive dehalogenase gene cluster, a two-component regulator system is encoded. Each of them includes a putative histidine-protein kinase (HPK; SMUL_1534 and SMUL_1538) and a putative response regulator (RR; SMUL_1535 and SMUL_1539). The HPK SMUL_1534 was identified only in Fo/Ni-ME, while the HPK protein SMUL_1538 was not detected. The RR protein SMUL_1539 was identified in Py/Fu-SF and quantified (7.4 to 7.6) in Py/PCE-SF, Py/Ni-SF and Fo/PCE-SF (Fig. 3). The RR protein SMUL_1535 could not be identified.

### Corrinoid biosynthesis

PceA harbors a corrinoid cofactor shown to be essential for the dehalogenation process^16^. The corrinoid cofactor of PceA is a norpseudo-B_12_^9^ and synthesized *de novo* by *S. multivorans*^10^. The genes encoding the proteins necessary for corrinoid biosynthesis are located in the OHR region downstream of the reductive dehalogenase gene clusters and the quinol dehydrogenase genes^6^. All proteins which are expected to be part of the corrinoid biosynthesis machinery were identified and quantified in the proteome of *S. multivorans*, with the exception of CobS (cobalamin 5′-phosphate synthase, SMUL_1549) and CbiJ (cobalt-precorrin-6x reductase, SMUL_1564). CobS is predicted to be membrane-integral through seven transmembrane helices, which might be the reason for the apparent absence of the protein in the proteome. CbiB (adenosylcobinamide-phosphate synthase, SMUL_1543), which is predicted to contain six transmembrane helices, was identified only in Py/PCE-ME. Almost all proteins of the corrinoid biosynthesis identified in this study were exclusively detected in PCE-grown cells or their levels were at least significantly higher in the presence of PCE (Fig. 3, Tables S2 and S3). Seventeen of the 20 identified proteins of the corrinoid biosynthesis cluster could be quantified in at least one sample of PCE-grown cells. The proteins CobC (alpha-ribazole phosphatase, SMUL_1550) and CbiG (cobalamin biosynthesis protein, SMUL_1561) could be identified only in Py/PCE-SF. In general, proteins detected in a lower amount or identified once or not at all, are involved in corrin ring biosynthesis and modification (CbiD, CbiE, CbiA, CbiG, CbiJ). A unique enzyme putatively involved in *de novo* corrinoid biosynthesis of *S. multivorans* is SMUL_1544, which displays very low sequence identity to biochemically characterized threonine phosphate decarboxylases (CobD)^6^. This protein might be responsible for the production of ethanolamine phosphate, which may be incorporated as linker moiety into the norpseudo-B_12_’s nucleotide loop. It was detected in the soluble fractions of all PCE-grown cells in a significant amount (8.8 to 9.0) at 2 to 5-times higher levels than in the membrane extracts. Of the corrinoid ABC transporter BtuCDF, only the periplasmic component BtuF was detected. Neither BtuC nor BtuD was detected, which might be due to their tight interaction with the membrane.

Several proteins encoded in the corrinoid biosynthesis cluster are not assigned to any function in corrinoid biosynthesis. The gene products of SMUL_1548 and SMUL_1567 are 10 and 8 kDa large proteins, containing eight cysteines each. Similar proteins (approximately 50% amino acid sequence identity) are found in a range of bacteria, primarily Fusobacteria, Firmicutes and Delta-, Epsilon- and Gammaproteobacteria. The gene product of SMUL_1548 was found in Py/PCE-SF and Fo/PCE-SF, while that of SMUL_1567 was not detected in any sample. The gene product of SMUL_1565, an MsbA-like protein, was only identified in Py/Fu-grown cells of *S. multivorans*.

The TetR-like transcriptional regulator downstream of the corrinoid biosynthesis cluster (SMUL_1569 and 1572) was not identified in any of the samples, which is in line with the observation that the corresponding gene is disrupted by a transposase (SMUL_1570 and 1571). Downstream of the corrinoid biosynthesis cluster and the *tetR* pseudogene, several genes are located encoding putative proteins for which participation in corrinoid biosynthesis is unproven. One of these proteins (SMUL_1573) was detected in PCE-grown cells only, while the other one (SMUL_1575, a putative FMN-binding protein) is found at >50-fold higher levels in PCE-grown cells than with fumarate or nitrate, regardless of the electron donor. Since close relatives of both proteins are found in *Ilyobacter polytropus*, which has a similar corrinoid biosynthesis cluster^6^,^17^, these proteins might have a role in corrinoid biosynthesis in *S. multivorans*. The two other proteins (SMUL_1574, a putative FeS cluster and FMN containing protein, and SMUL_1576, a putative membrane protein) were not detected. The proteins involved in the biosynthesis of uroporphyrinogen III, the precursor for biosynthesis of corrinoids (SMUL_0533, SMUL_1083, SMUL_1902 and 1906), are not differentially regulated with PCE as electron acceptor. Interestingly, HemD (SMUL_0775) (uroporphyrinogen-III synthase) was quantified only with nitrate as electron acceptor.

### PCE-induced proteins encoded outside the OHR region

Only few proteins encoded outside the OHR region were more abundant in cells grown with PCE compared to fumarate or nitrate. These proteins are encoded at different locations throughout the whole genome, but perform related biological functions. Several putative chaperones are among the most abundant proteins in PCE-grown cells (Table S4) but not in cells grown with other electron acceptors. Heat shock protein Hsp20 (SMUL_0547) was quantified at approx 20 to 60 times higher levels in all PCE-samples (Table S3), making it the second most abundant protein in Py/PCE-SF (Table S4). Chaperone HtpG (SMUL_ 2014) was detected in higher levels in PCE-grown cells as well (between 3 and 7-fold). This is in contrast to HtpG and Hsp20 in dechlorinating *Desulfitobacterium* spp^18^,^19^, where instead chaperones GroEL/ES are present in a higher amount in organohalide-grown cells. The latter chaperones were quantified in similar amounts in all samples of *S. multivorans*. Several other stress-related proteins are also more abundant in PCE-grown cells. The 11^th^ most abundant proteins in Fo/PCE-SF is a superoxide dismutase (SMUL_0529), present at elevated concentrations in all PCE-samples (3.1–7.4-fold). A glutathione peroxidase (SMUL_368), rarely encoded in epsilonproteobacterial genomes and closely related to the corresponding *Bacillus*-like enzyme, was only quantified in PCE-grown cells (Py/PCE-ME and Fo/PCE-SF). A number of flagellar proteins showed higher levels after PCE cultivation. Flagellin (SMUL_3194; 5.6 to 72-fold, p < 0.05) and the flagellar hook-associated protein FlgL (SMUL_1598; up to >50-fold, p < 0.01) were present at significantly altered levels (Table S3). Furthermore, FliA, the flagellar sigma factor (SMUL_0381) was found in higher levels (up to 7.3-fold) in Fo/PCE cells and was not quantified in Py/Fu or Py/Ni-grown cells.

### Oxidative catabolism

Electron donors used for growth of *S. multivorans* in this study were formate and pyruvate. Formate is oxidized via a membrane-bound, molybdopterin-containing formate dehydrogenase (Fdh) not encoded in the OHR region^6^,^20^. The Fdh2 subunits FdhA2 (SMUL_2873) and FdhB2 (SMUL_2872) were quantified in high amounts in both, pyruvate (8.0 to 8.9 for FdhA2 in the ME) and formate-grown cells (9.1 to 9.3, Table S1). The membrane-integral cytochrome *b* subunit FdhI2 (SMUL_2871) was identified only in Fo/PCE-ME. The formate dehydrogenase-specific chaperon FdhX1 (SMUL_2875) and the formate dehydrogenase accessory protein FdhD (SMUL_2870) could be quantified in samples obtained from all conditions. Whereas FdhD is present in medium to high amounts (7.8 to 8.9) and the protein is more abundant in formate-grown cells, FdhX1 is present in low to medium amounts (6.3 to 8.0) and found to be present in higher levels in pyruvate-grown cells (Table S1). Furthermore, two additional formate dehydrogenases (Fdh1, SMUL_970 to 972 and Fdh3, SMUL_2899 to 2901) were quantified in Fo/Fu-grown cells, where a significant amount (8.4 and 8.1, respectively, for the catalytic subunits) was detected. Additionally, gene products of Fdh1 were identified in Py/PCE and Py/Ni-grown cells. A cytoplasmic formate dehydrogenase encoded by SMUL_0079 was quantified as a protein with low abundance (6.7 to 7.4) under all growth conditions applied (Table S1).

Besides formate, hydrogen can be used as electron donor by *S. multivorans*. The genome of *S. multivorans* harbors four gene clusters encoding NiFe hydrogenases, two of which can be classified as hydrogen-uptake hydrogenases^6^. Even though hydrogen was not chosen as a growth substrate in this study, a membrane-bound hydrogen-uptake hydrogenase (SMUL_1423 to 1425, MBH) was detected in high amounts (large subunit values ranging from 9.2 to 9.9 in the membrane extract) in all samples. Moreover, all accessory proteins required for the maturation of the Ni-Fe active site (namely HypBCDE) were found with the exception of HypA. The three other hydrogenases encoded in the genome of *S. multivorans* were either detected in only a part of the samples or not identified at all. A putatively hydrogen-evolving hydrogenase of *S. multivorans* (encoded by SMUL_2383 to 2392), similar to hydrogenase 3 (Hyc) and 4 (Hyf) as part of the formate hydrogen lyase of *E. coli*^21^, was detected and quantified preferentially in cells grown with pyruvate as electron donor (Table S1). The second uptake hydrogenase was not detected at all, while only one subunit (out of four) of a second putative hydrogen-evolving hydrogenase was quantified in Fo/PCE cells exclusively.

For the oxidation of pyruvate as electron donor, two different enzymes were detected in this study, of which the pyruvate:ferredoxin/flavodoxin oxidoreductase (PFOR) was more abundant under all conditions. The PFOR was detected at increased concentrations (1.2 to 7.5-fold) in all fractions from cells grown with pyruvate compared to formate. The electron acceptor for pyruvate oxidation mediated by PFOR of *S. multivorans* might be either ferredoxin or flavodoxin^6^. Here, only one ferredoxin (encoded by SMUL_303) was more abundant in pyruvate-grown cells compared to formate (up to 6-times), while no flavodoxin was quantified in any of the samples (Table S1). The second pyruvate-oxidizing enzyme, pyruvate dehydrogenase (quinone), PoxB (SMUL_1703), was quantified in all samples of cells grown with pyruvate as electron donor. With formate as electron donor, it was only quantified in Fo/PCE-ME and Fo/Ni-SF (Table S1). Remarkably, *poxB* is exclusively found in *S. multivorans* among all Epsilonproteobacteria sequenced up to date (Genbank nr database as of June 2015) and is closely related (58% amino acid sequence identity) to the enzyme in *E. coli*^22^.

*S. multivorans* encodes two different respiratory complex I forms on its genome. A typical (“standard”) complex I (Nuo, encoded by SMUL_0508 to 0520) as found in most bacteria (e.g. *E. coli*) and another one specific for Epsilonproteobacteria (encoded by SMUL_0195 to 0208). The main difference between both complexes is the function of two cytoplasmic subunits (NuoEF or NuoEF-like proteins), which interact with NAD^+^ in the former and with either ferredoxin or flavodoxin in the latter^23^. While up to eight subunits of the epsilonproteobacterial-type could be detected and maximally seven of them quantified, only one to four subunits of the “standard” complex I were identified, whereas in most conditions none of them were quantified (Table S1).

All enzymes of the ε-proteobacterial TCA cycle present in *Campylobacter jejuni*^24^ were detected in medium to major amounts (approximately 8 to 10) under all conditions applied (Table S1). Malate is an important intermediate of the TCA cycle and numerous enzymes that can produce or oxidize malate are encoded in the genome of *S. multivorans*. They include two malate dehydrogenases (SMUL_0065 and 1443), a malate synthase (SMUL_0148), a malic enzyme (reversibly decarboxylating malate to pyruvate, SMUL_3158) and a malate-quinone oxidoreductase (SMUL_0667). Of these four proteins, the malate-quinone oxidoreductase is detected in major amounts in the membrane extract in all samples (8.7 to 9.2) and the malic enzyme in both, membrane and soluble extract (9.1 to 9.8). Of the two malate dehydrogenases (NAD^+^-reducing), only SMUL_1443 was quantified in low to medium amounts (7.5 to 7.9). The malate synthase was quantified (7.2 to 7.8) in all pyruvate-grown cells, and in Fo/PCE-SF.

### Nitrate respiration

The nitrate reductase operon is encoded by SMUL_0934 to 0940 (NapAGHBLD) and the ammonifying nitrite reductase operon by SMUL_0889 to 0892 (NrfHAIJ). Of the latter, only NrfA and NrfJ were identified or quantified. Both proteins were detected in most of the samples, regardless of the electron acceptor, although NrfJ was not identified in Fo/Ni-grown cells. In the latter cells, the catalytic subunit NrfA was detected in much higher amounts (six to >50 times) than in cells grown with PCE or fumarate as electron acceptor, whereas the difference in pyruvate-grown cells was negligible. The nitrate reductase catalytic subunit NapA (SMUL_0934) and cytochrome subunit NapB (SMUL_0937) were present in a higher amount in all nitrate-grown cells (3 to >50-times compared to PCE- or fumarate-grown cells). The periplasmic subunit NapG of the quinol dehydrogenase was quantified in Py/Ni-SF but not detected in any of the pyruvate-grown cells without nitrate. In Fo/Ni-grown cells, NapG was more abundant (7 to 9-fold ratio) and NapH could be quantified in the membrane extract, opposed to the membrane-integral subunit of the PCE-induced quinol dehydrogenase (see above). One protein which is drastically more abundant in all nitrate-grown cells is a hydroxylamine reductase (SMUL_0602). While not identified in any of the cells grown without nitrate, it was among the most abundant proteins in both, Fo/Ni and Py/Ni-grown cells, where the amount was two to seven times higher in the soluble fraction than in the membrane extract.

## Discussion

The differential proteomic analysis of *S. multivorans* cells grown under different substrate combinations allowed for the identification of components that might play a role in organohalide respiration of Epsilonproteobacteria including norpseudo-B_12_ biosynthesis, and it gives a comprehensive view on the basic catabolism of this free-living Epsilonproteobacterium.

The identification of 53% of the gene products annotated for *S. multivorans* provides a solid basis for comparative analysis of the different growth conditions. A large proportion of undetected proteins in this approach might not be synthesized as they may be required exclusively under different physiological conditions or during growth on other substrates.

### Organohalide respiration and PCE stress response

The most remarkable differences in the analyzed proteomes of cells grown with and without PCE could be assigned to the OHR core region, which contains genes linked to organohalide respiration. Genes up- and downstream of this core are not induced with PCE, demonstrating that they do not play a role in PCE metabolism. The long-term down-regulation of PceA^6^,^12^ is supported by the presented results.

Corrinoid biosynthesis proteins detected in this study were predominantly found in PCE-grown cells (Fig. 3), as opposed to *Desulfitobacterium hafniense* or *D. dehalogenans*, where an organohalide-dependent increased amount of these proteins has not been detected^19^, except for CobT of *D. hafniense* TCE1 grown with PCE^18^. In *S. multivorans*, the norpseudo-B_12_ cofactor seems to be exclusively required for PceA synthesis under the tested conditions. The unique structural feature of the norpseudo-B_12_ produced by *S. multivorans* is the absence of methyl group 176 in the linker moiety of the nucleotide loop^9^. The *S. multivorans* enzyme involved in the nucleotide loop assembly pathway and most probably responsible for this difference is a distant homologue of CobD (SMUL_1544). Indeed, this enzyme was found in the proteome only in cells grown with PCE, further strengthening its role in production of norpseudo-B_12_. Several proteins encoded in or downstream of the corrinoid biosynthesis gene cluster lack an assigned function. The cysteine-rich proteins SMUL_1548 and SMUL_1567 might be redox-active proteins through metal-binding or disulfide bridge formation and may aid processes in corrinoid biosynthesis. The PCE-dependent high abundance of SMUL_1573 and SMUL_1575, two presumably flavin-containing proteins of unknown function encoded by genes downstream of the corrinoid biosynthesis cluster is remarkable. It is feasible that flavin-containing enzymes help to sustain low redox states of the central cobalt during corrinoid biosynthesis^25^. PceB was found exclusively in membrane extracts of PCE grown cells, supporting the assumption that PceB is the membrane anchor of the PCE reductive dehalogenase^8^ and that it is required for PCE respiration in *S. multivorans*. A second reductive dehalogenase, RdhA is encoded in the OHR region (SMUL_1536) and was identified only in formate/PCE-grown cells. The results clearly show that *rdhAB* is not induced with PCE as electron acceptor in general. Hence, the physiological function of this second reductive dehalogenase remains enigmatic.

In the OHR region, two genes (SMUL_1541 and SMUL_1542) encode a putative membrane-bound, periplasmic quinol dehydrogenase (Qdh)^6^, of which SMUL_1541 was detected exclusively in PCE-grown cells. This points towards a participation of this Qdh in the PCE respiratory chain, transferring electrons from the menaquinone pool to PceA (Fig. 4), analogous to the role of NapGH in nitrate respiration^6^,^11^,^26^. Despite containing no membrane helices, SMUL_1541 was present exclusively in the membrane extract. This is likely due to a close interaction with the membrane-integral subunit of the Qdh (SMUL_1542), which was not detected in any sample, despite the previous detection of the corresponding mRNA in PCE-grown cells^6^. SMUL_1542, predicted to contain four membrane helices, was apparently not extracted from the membrane in this study. Candidates for the regulation of PCE respiration are the two-component regulatory systems downstream of each *rdh* cluster. Quantification of the response regulator encoded by SMUL_1539, downstream of the *rdhAB* cluster, indicates a participation of this protein in up-regulation of genes included in the OHR “core” region. This regulator was also identified and quantified in cells cultivated without PCE, which is in accordance with the putative function of SMUL_1539 in PCE-sensing, an ability that has to be maintained especially in the absence of PCE.

An alkylhydroperoxidase-like protein (SMUL_1530) was detected exclusively in PCE-grown cells. The protein is related (around 60% amino acid sequence identity) to a group of uncharacterized AhpD alkylhydroperoxidase-family proteins found in several other free-living, often marine Epsilonproteobacteria, Gammaproteobacteria, and Aquificales. The similarity to the characterized *Mycobacterium tuberculosis* alkylhydroperoxidase AhpD is low (~10%), but the catalytic residues [CxxCxxxH] are conserved^27^. As AhpD is a part of oxidative stress response in the latter bacterium, SMUL_1530 could function to protect proteins required in PCE metabolism from oxidative stress. In the proteome of the aerobic cDCE-degrading bacterium *Polaromonas* sp. JS666, an AhpC-like peroxidase was found in a higher amount in cells grown with cDCE^28^. As reductive (anaerobic) dechlorination is highly unlikely to be carried out by this organism, a role of peroxidases in the general stress response to chlorinated ethenes seems feasible. A protein similar to IscU and NifU^29^,^30^ (SMUL_1533) might have a function in the correct insertion of the FeS clusters in PceA^31^. Both proteins, the products of SMUL_1530 and 1533, are not found in other organohalide-respiring bacteria, therefore, their occurrence in *S. multivorans* may indicate a special role in PceA maturation in this organism.

Outside the OHR region, several proteins presumably involved in stress response were quantified in a larger amount in PCE-grown cells. This is in accordance with the fact that polychlorinated ethenes are toxic at elevated concentrations due to their high hydrophobicity. A non-characterized protein belonging to the Hsp20 heat shock family proteins (SMUL_0547), containing the so-called alpha-crystallin domain, was detected in higher amounts in PCE-grown cells. This protein family has been suggested to act ATP-independently as a multimeric chaperone, aiding in refolding proteins especially under stress^32^,^33^. A similar role might be fulfilled by HtpG, which was shown to have protein refolding activity in *E. coli*^34^. The higher abundance of a superoxide dismutase and glutathione peroxidase in PCE-grown cells is difficult to explain, as these are radical scavenging enzymes often involved in detoxifying reactive oxygen species^35^,^36^. However, it is assumed that superoxide dismutase may be also part of the general stress response mechanism as described for *B. subtilis*^37^. In *D. hafniense* TCE1, a catalase was found in higher amounts in PCE-grown cells^18^, which might have a similar role in this organism. In PCE-grown cells of *S. multivorans*, these enzymes might be expressed either to detoxify radical intermediates formed in the process of reductive dechlorination^38^,^39^ or as a component of the global stress response. The higher abundance of flagellar proteins, especially flagellin, might also be a part of the general stress response in *S. multivorans* or, alternatively, due to damage of the flagellum by chlorinated ethenes.

### Oxidative catabolism

When formate serves as electron donor in *S. multivorans*, it is oxidized in the periplasm by a formate dehydrogenase, which donates electrons via a membrane-integral cytochrome *b* to the menaquinone pool. Therefore, the combination of formate as electron donor and PCE or nitrate as electron acceptor leads to a typical membrane-located respiratory chain (Fig. 4A). Of the three membrane-bound formate dehydrogenases encoded in the genome of *S. multivorans*^6^, most probably Fdh2 (SMUL_2871 to 2873) is the main enzyme complex involved in respiratory formate oxidation^20^. The other two formate dehydrogenases seem to fulfill a backup formate oxidation system, since they were detected only in cells grown with formate and fumarate. The fact that Fdh2 is present in pyruvate-grown cells might be due to a constitutive expression. This might also apply to the membrane-bound hydrogenase (MBH), since the latter was detected under all growth conditions in high amounts. A similar situation was observed in several *Desulfitobacterium* spp.^19^,^40^. The constitutive expression of Fdh2 and MBH points towards the importance of both formate and hydrogen as electron donors in *S. multivorans* which might be among the preferred available electron donors in natural habitats of *Sulfurospirillum* spp. such as sludge or sediments. The second hydrogenase which was quantified in high amounts is the presumably hydrogen-evolving Hyf hydrogenase. It might function as an electron sink for excess reduced ferredoxin produced by PFOR in case of electron acceptor limitation. Additionally, the presence of a hydrogen-producing enzyme may explain the high amount of MBH which is possibly responsible for hydrogen recycling.

The route of electrons generated by pyruvate oxidation to the terminal electron acceptor is not clear in *S. multivorans*. Pyruvate could be oxidized via two enzymes. One of them is the typical epsilonproteobacterial PFOR, which most likely reduces a ferredoxin encoded by SMUL_0303. This is opposed to the situation in the Epsilonproteobacterium *Helicobacter pylori*, where presumably flavodoxin is interacting with the PFOR^41^. An ortholog of this flavodoxin (SMUL_2785) was not detected in this study. In the Firmicutes *D. hafniense* Y51, PFOR was also suggested to transfer electrons from pyruvate to ferredoxin^40^. In *S. multivorans*, the reduced ferredoxin could be oxidized by the epsilonproteobacterial complex I, which then transfers electrons to the quinone pool (Fig. 4B). The second enzyme used for pyruvate oxidation by *S. multivorans* is pyruvate (quinol) dehydrogenase, a cytoplasmic lipoenzyme which interacts directly with the quinone pool^22^,^42^. This enzyme seems to be mainly synthesized in pyruvate-grown cells, but as high amounts of the PFOR are present in cells grown with pyruvate as electron donor, both enzymes could be responsible for pyruvate oxidation (Fig. 4B).

### Nitrate respiration

The response of *S. multivorans* to nitrate as electron acceptor is of interest, since nitrate respiration parallels PCE respiration in the way that both systems contain a periplasmic terminal reductase and most probably involve a quinol dehydrogenase. Although single steps of respiratory nitrate ammonification and the intermediates formed have not yet been determined for *S. multivorans*, we have been able to identify enzymes and reactions likely involved in this pathway by comparative proteome analysis of cells grown with nitrate, PCE, or fumarate. The higher abundance of the nitrate reductase NapAB and the quinol dehydrogenase NapGH in nitrate-grown cells points towards a nitrate-dependent regulation of nitrate respiratory chain proteins. However, the high amount of NapAB in formate-grown cells without nitrate indicates a global regulation of nitrate respiratory enzymes dependent on the carbon and energy source. Highly significant is the large amount of a hydroxylamine reductase (hybrid cluster protein) in all nitrate-grown cells. Hydroxylamine is presumably not released as reaction intermediate during nitrate ammonification in contrast to denitrification^43^ nor is hydroxylamine reductase part of the stress response to hydroxylamine in the closely related *Wolinella succinogenes*^44^. Recently, an enzyme complex was reported to be responsible for hydroxylamine production in the Epsilonproteobacterium *Nautilia profundicula*^45^, but the central enzyme of this complex (the periplasmic hydroxylamine oxidoreductase) is not encoded in the genome of *S. multivorans*. However, the hydroxylamine reductase of *S. multivorans* might function as scavenger of other intermediates of nitrate ammonification such as nitrite or NO as reported for other bacteria^46^,^47^. Therefore, it can be assumed that the increased level of the hydroxylamine reductase is part of a global response to the presence of nitrate.

## Conclusion

This proteome analysis sheds light on components linked to the organohalide-respiratory chain, proteins involved in maturation thereof and the global response to PCE and other energy substrates in the Epsilonproteobacterium *S. multivorans*. A NapGH-like quinol dehydrogenase was identified as the most probable link between the quinone pool and the reductive dehalogenase PceA. In this respect, PCE respiration resembles Nap-mediated nitrate respiration. A NifU/IscU-like protein not observed in other organohalide-respiring bacteria before might be specific for FeS cluster assembly of PceA. Detection of the norpseudo-B_12_ biosynthesis machinery exclusively in PCE-grown cells confirms the crucial role of the corrinoid cofactor for PCE respiration. A peroxidase-like protein might protect PceA against oxidative stress. The global response to PCE involves an increase in the production of chaperones, which differ from those found in dechlorinating *Desulfitobacterium* spp. This study also provides first insights into the electron transfer from pyruvate to PCE including the potential involvement of a quinone-reducing pyruvate dehydrogenase and of a pyruvate:ferredoxin/flavodoxin oxidoreductase presumably reducing the ferredoxin SMUL_0303. An epsilonproteobacterial complex I mediates the electron transfer from ferredoxin to the quinone pool.

All in all, these findings contribute to a deeper insight into organohalide respiration and might help to better understand the general bacterial response to these widely distributed but environmentally harmful substances. Furthermore, this proteome analysis might aid the research on the hitherto underexplored ecophysiology of free-living Epsilonproteobacteria.

## Methods

### Cultivation of *S. multivorans*

*S. multivorans* (DSMZ 12446) was cultivated under anaerobic conditions at 28 °C in a defined mineral medium^4^ without vitamin B_12_ (cyanocobalamin). Pyruvate (40 mM) or formate (40 mM) were used as electron donor and fumarate (40 mM), nitrate (10 mM with formate, 40 mM with pyruvate as electron donor) or PCE as electron acceptor. PCE was added to the medium (10 mM nominal concentration) from a hexadecane stock solution (0.5 M). When the cells were grown with formate, acetate (5 mM) was added as carbon source and the medium contained 0.05% yeast extract to support growth. Titanium(III)-citrate (5.6 μM) was added when *S. multivorans* was grown with formate and nitrate (10 mM). Cultivation of *S. multivorans* was performed using the following substrate combinations: pyruvate/fumarate (Py/Fu), pyruvate/PCE (Py/PCE), pyruvate/nitrate (Py/Ni), formate/fumarate (Fo/Fu), formate/PCE (Fo/PCE) and formate/nitrate (Fo/Ni). Pre-cultures were grown in rubber-stoppered 200 mL glass serum bottles and the main cultures in rubber-stoppered 2 L glass bottles. The ratio of aqueous to gas phase was always 1:1. In order to generate *S. multivorans* cells with down-regulated *pceA* gene expression^12^, the organism was cultivated for 60 subcultivation steps (10% inoculum, each) on pyruvate (40 mM) and fumarate (40 mM) plus 0.2% yeast extract. The last culture was used as inoculum for all pre-cultures. The inoculum corresponded to approximately 18 μg/ml cell protein for Py/Fu-grown cells, 12 μg/ml for Fo/Fu cells, 6 μg/ml for Py/PCE and Py/Ni-grown cells and 3 μg/ml cell protein in the case of Fo/Ni and Fo/PCE cultures. The bacterial growth was monitored photometrically by measuring the optical density at 578 nm. All cultivations were performed in triplicates.

### Cell harvesting and samples preparation

*S. multivorans* cells were harvested in the late exponential growth phase by centrifugation (12,000 × g, 10 min at 10 °C). The cell pellets were washed once in 50 mM Tris-HCl (pH 7.5) and resuspended (1:2) in the same buffer containing a tip of a spatula of DNase I (AppliChem, Darmstadt, Germany) and protease inhibitor (one tablet for 10 ml buffer; cOmplete Mini, EDTA-free; Roche, Mannheim, Germany). The cells were disrupted using a French Press (1000 psi). Cell debris was removed by centrifugation (6,000 × g, 10 min at 4 °C). The supernatant was ultracentrifuged (260,000 × g, 45 min at 4 °C) and the resulting supernatants were decanted (soluble protein fractions = SF). The pellets (membrane extract) were washed twice with 50 mM Tris-HCl (pH 7.5) including protease inhibitor and finally resuspended in solubilization buffer (1% (w/v) Digitonin (AppliChem, Darmstadt, Germany), 300 mM NaCl, 100 mM Tris-HCl, pH 7.5). The membrane protein extracts were obtained by stirring overnight at 16 °C. After ultracentrifugation (260,000 × g rpm, 45 min at 4 °C), the supernatants (membrane protein extracts = ME) were collected and analyzed (Fig. 1).

### Immunoblot analysis

Soluble fractions (10 μg protein per lane) were subjected to denaturing SDS PAGE (12.5%) and afterwards blotted onto a polyvinylidene difluoride (PVDF) membrane (Roche, Mannheim, Germany) using a semi-dry transfer cell (Bio-Rad, Munich, Germany) according to the protocol described by John *et al.* (2009). The PceA antiserum (primary antibody) was diluted 500,000-fold. The primary antibody was detected via a secondary antibody (diluted 1:20,000) coupled to alkaline phosphatase (Sigma-Aldrich, Munich, Germany).

### Measurement of PceA activity

*S. multivorans* cells were harvested in the late exponential growth phase by centrifugation (12,000 × g, 10 min at 10 °C) under air. Cell pellets were washed with 50 mM Tris-HCl (pH 7.5). The cell pellets were transferred into an anoxic glove box and resuspended (1:2) in anoxic buffer (50 mM Tris-HCl, pH 7.5). An equal volume of glass beads (0.25–0.5 mm diameter, Carl Roth GmbH, Karlsruhe, Germany) was added and the cells were disrupted using a bead mill (5 min at 25 Hz; MixerMill MM400, Retsch GmbH, Haan, Germany). The crude extracts were separated from the glass beads by centrifugation (14,000 × g, 2 min) under anoxic conditions. The measurements of PceA activity were performed as described before using a photometric assay with reduced methyl viologen as artificial electron donor^7^.

### SDS-PAGE and proteolytic digestion

The protein concentration was determined after protein extraction using the Bio-Rad Bradford reagent (Bio-Rad, Munich, Germany) and bovine serum albumin as protein standard. Protein samples of solubilized membrane extract and soluble fractions were subjected to SDS-PAGE. Per lane, 50 μg of protein lysates was applied. Afterwards, each sample lane was cut into four slices and prepared for proteolytic cleavage using trypsin (Promega, Madison, WI, USA)^48^. Peptide lysates were extracted and desalted using C18 ZipTips (Merck Millipore, Darmstadt, Germany).

### Mass spectrometry and proteome data analysis

Separation of tryptic peptides was performed using an Ultimate 3000 nanoRSLC system (Thermo Scientific, Germering, Germany) coupled to an Orbitrap Fusion mass spectrometer (Thermo Scientific, San Jose, CA, USA). A sample volume of 1 μL was loaded onto a trapping column with 300 μm inner diameter, packed with 5 μm C18 particles (μ-precolumn, Thermo Scientific) and separated via a 15 cm analytical column (Acclaim PepMap RSLC, 2 μm C18 particles, Thermo Scientific). The column oven temperature was set to 35 °C. During the liquid chromatography (LC) run, a constant flow of 300 nL/min (solvent A: 0.1% formic acid) was applied with a linear gradient of 4% to 55% solvent B (80% acetonitrile, 0.08% formic acid) in 90 min. Mass spectrometer (MS) full scans were measured in the Orbitrap mass analyzer within the mass range of 400–1,700 *m/z* at 60,000 resolution using an automatic gain control target of 4 × 10^5^ and maximum fill time of 50 ms. The MS analyzed in data-dependent acquisition mode; the highest intense ions with positive charge states between 2 and 7 were selected for MS/MS. An MS/MS isolation window for ions in the quadrupole was set to 1.6 *m/z*. MS/MS scan were acquired within 3 s of cycle time (Top Speed) using the higher energy dissociation mode at normalized collision induced energy of 35%, activation time of 120 ms, and minimum of ion signal threshold for MS/MS of 5 × 10^4^ counts. The exclusion time to reject masses from repetitive MS/MS fragmentation was set to 30 s.

LC-MS/MS data were analyzed using Proteome Discoverer (v1.4.1.14, Thermo Scientific). MS/MS spectra were searched against the data of the *S. multivorans* database containing 3,191 non-redundant protein-coding sequence entries (downloaded February 17^th^ 2014 from NCBI Genbank accession number CP007201) using the SEQUEST HT and MS Amanda algorithms with the following settings: trypsin as cleavage enzyme, oxidation of methionine as dynamic and carbamidomethylation of cysteine as static modification, up to two missed cleavages, MS mass tolerance set to 10 ppm and MS/MS mass tolerance to 0.05 Da, respectively. False discovery rate for peptides was <0.01 (Supplement information Table S1). Quantification of proteins was performed using the average of top 3 peptide area^49^. Protein quantification was considered successful for proteins quantified in >50% of biological replicates, otherwise they were classified as identified proteins. After log10 transformation, the protein values were normalized and bioinformatic analysis was applied by principal component analysis and T-test statistics (R). Significance threshold p < 0.05 in a two-tailed test were considered as significantly altered.

## Additional Information

**How to cite this article**: Goris, T. *et al.* Proteomics of the organohalide-respiring Epsilonproteobacterium *Sulfurospirillum multivorans* adapted to tetrachloroethene and other energy substrates. *Sci. Rep.* **5**, 13794; doi: 10.1038/srep13794 (2015).

## Supplementary Material

Supplementary Information

Supplementary Dataset 1

Supplementary Dataset 2

Supplementary Dataset 3

Supplementary Dataset 4
