# Supplementary material for: Proteomics of the organohalide-respiring Epsilonproteobacterium Sulfurospirillum multivorans adapted to tetrachloroethene and other energy substrates
Source: Sci Rep. 2015 Sep 21;5:13794. doi: 10.1038/srep13794 (PMC4585668; doi:10.1038/srep13794)
Supplement: Supplementary Information [file srep13794-s1.pdf]

Supplemental material to:

Proteomics of the organohalide-respiring Epsilonproteobacterium *Sulfurospirillum multivorans* adapted to tetrachloroethene and other energy substrates

Tobias Goris[*,§,1], Christian L. Schiffmann[§,2], Jennifer Gadkari[§,1], Torsten Schubert[1], Jana Seifert[3], Nico Jehmlich[2], Martin von Bergen[2,4,5] Gabriele Diekert[1]

[§]authors contributed equally

[1]Department of Applied and Ecological Microbiology, Institute of Microbiology, Friedrich Schiller University, 07743 Jena, Germany

[2]Department of Proteomics, Helmholtz Centre for Environmental Research – UFZ, Permoserstr. 15, 04318 Leipzig, Germany

[3]Institute of Animal Science, Hohenheim University, 70593 Stuttgart, Germany

[4]Aalborg University, Department of Biotechnology, Chemistry and Environmental Engineering, Sohngårdsholmsvej 49, 9000 Aalborg, Denmark

[5]Department of Metabolomics, Helmholtz Centre for Environmental Research – UFZ, Permoserstr. 15, 04318 Leipzig, Germany

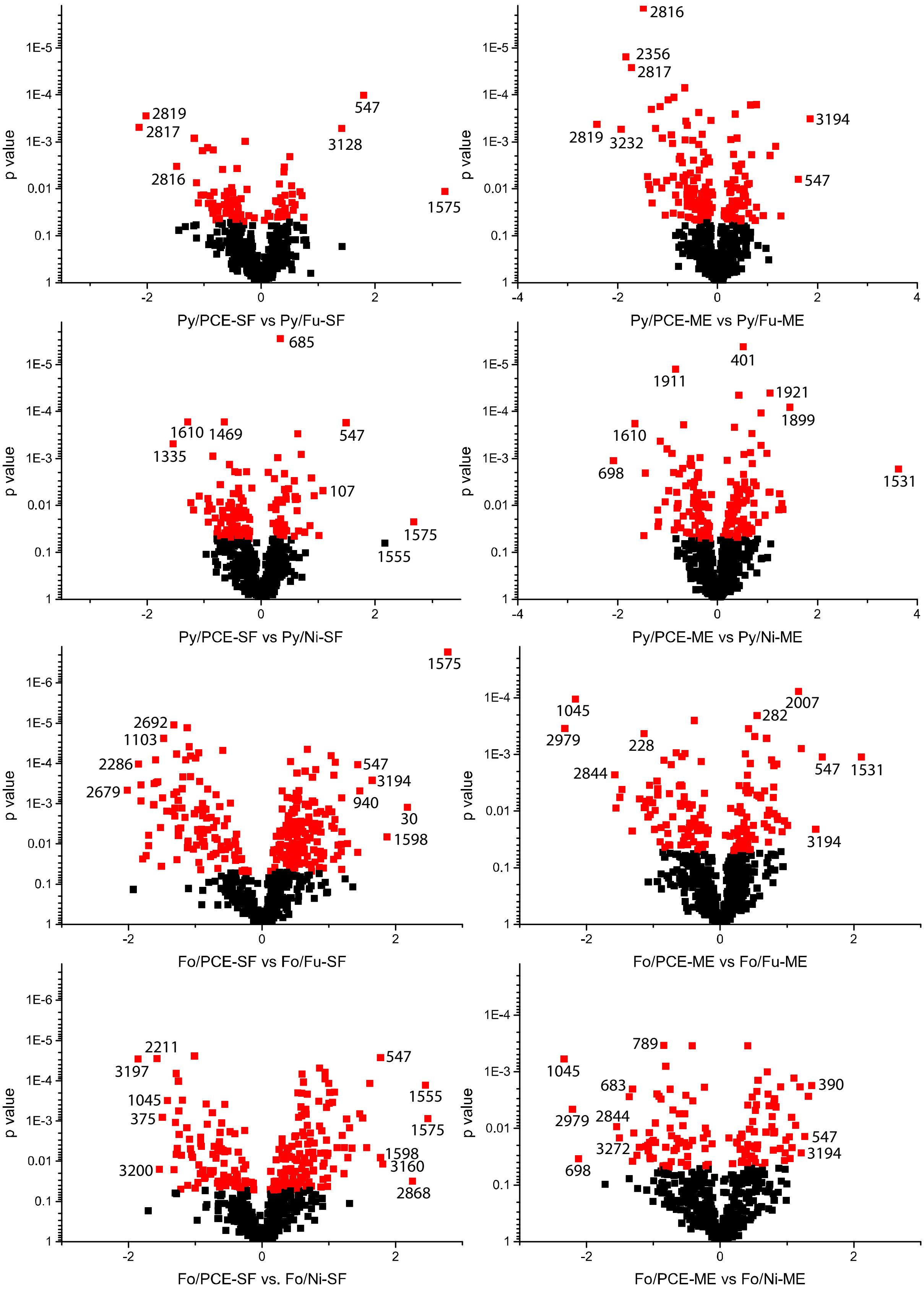

Fig. S1: Volcano plots for differential comparison of the quantified proteins (Visualization of Table S3). The x-axis shows the difference in the protein amount as calculated and normalized from the top 3 peptide area (as described in materials and methods). Values are shown as log10. A value of more than 0.477 is taken as significant (equals an approximately 3-fold amount). The y-axis shows statistical significance as a result from the T-test. A p-value of less than 0.05 is taken as significant (marked in red). Numbers of the SMUL_locus tags are given for the proteins with the most significant changes (see Table S3).

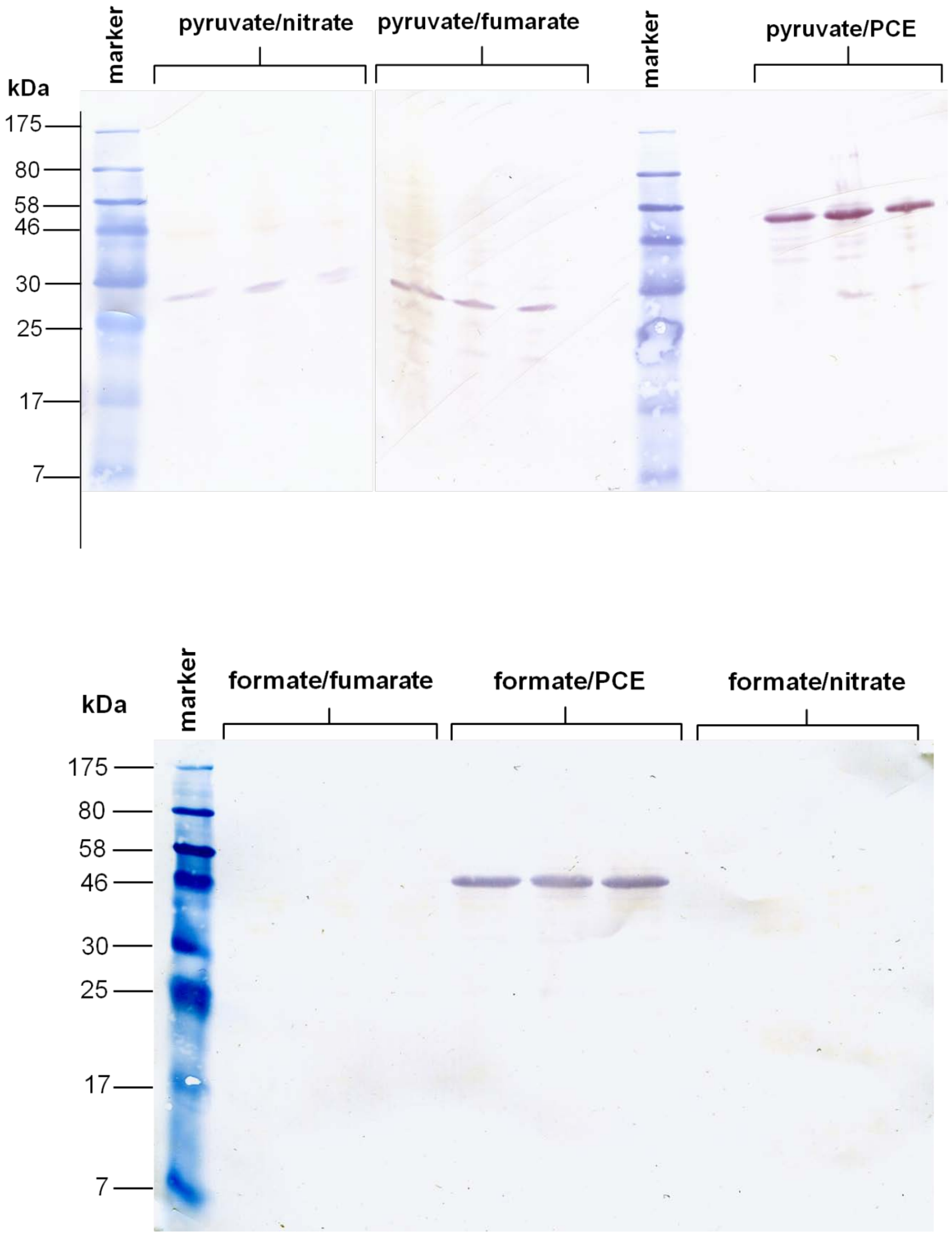

Fig. S2: Western-Blots: Anti-PceA (1:500.000). 10 µg protein from all biological replicates which were used in the proteome analysis was applied per lane. PceA is visible at approximately 50kDa. The band visible in samples from pyruvate-grown cells without PCE at around 27kDa is an unknown protein product, possibly degraded PceA. Blots were applied to tonal correction.

Table S5: PceA activity. The PceA activities were measured photometrically as described in the materials and methods section.

Sample	$Volume_{sample}$ [µl]	volume activity [nkat/ ml]	Specific activity [nkat/ ml]	GC-measurement (increase of cDCE)
**Pyruvate/Fumarate**	100	1,328	0,320	-
**Pyruvate/PCE**	5	213,752	20,183	++
**Pyruvate/Nitrate**	100	10,2067	1,180	-
**Formate/Fumarate**	100	3,9683	0,4663	+
**Formate/PCE**	5	75,9717	20,812	++
**Formate/Nitrate**	100	-	-	-
